# Acute oxalate nephropathy associated with orlistat

**DOI:** 10.15171/jnp.2016.14

**Published:** 2016-03-29

**Authors:** Youshay Humayun, Kenneth C. Ball, Jack R. Lewin, Anna A. Lerant, Tibor Fülöp

**Affiliations:** ^1^Department of Medicine, University of Mississippi Medical Center, Jackson, Mississippi, USA; ^2^Department of Pathology, University of Mississippi Medical Center, Jackson, Mississippi, USA; ^3^Department of Anesthesiology, University of Mississippi Medical Center, Jackson, Mississippi, USA; ^4^Simulation and Interprofessional Education Center, University of Mississippi Medical Center, Jackson, Mississippi, USA

**Keywords:** Acute kidney injury, Dialysis, Orlistat, Oxalate nephropathy, Proton-pump inhibitor, Weight loss supplement

## Abstract

*Background:* Obesity is a major world-wide epidemic which has led to a surge of various weight loss-inducing medical or surgical treatments. Orlistat is a gastrointestinal lipase inhibitor used as an adjunct treatment of obesity and type 2 diabetes mellitus to induce clinically significant weight loss via fat malabsorption.

*Case Presentation:* We describe a case of a 76-year-old female with past medical history of chronic kidney disease (baseline serum creatinine was 1.5-2.5 mg/dL), hypertension, gout and psoriatic arthritis, who was admitted for evaluation of elevated creatinine, peaking at 5.40 mg/dL. She was started on orlistat 120 mg three times a day six weeks earlier. Initial serologic work-up remained unremarkable. Percutaneous kidney biopsy revealed massive calcium oxalate crystal depositions with acute tubular necrosis and interstitial inflammation. Serum oxalate level returned elevated at 45 mm/l (normal <27). Timed 24-hour urine collection documented increased oxalate excretion repeatedly (54-96 mg/24 hour). After five renal dialysis sessions in eighth days she gradually regained her former baseline kidney function with creatinine around 2 mg/dL. Given coexisting proton-pump inhibitor therapy, only per os calcium-citrate provided effective intestinal oxalate chelation to control hyperoxaluria.

*Conclusions:* Our case underscores the potential of medically induced fat malabsorption to lead to an excessive oxalate absorption and acute kidney injury (AKI), especially in subjects with pre-existing renal impairment. Further, it emphasizes the importance of kidney biopsy to facilitate early diagnosis and treatment.

**Implication for health policy/practice/research/medical education:**

Acute oxalate nephropathy may be an under-recognized and important cause of renal failure in patients taking fat malabsorbtive weight loss supplements. Percutaneous kidney biopsy and timed 24-hour urine collections for oxalate excretion may expedite the diagnosis. The oxalate binding properties of per os calcium supplements are not sufficiently studied in advanced renal failure.

## 1. Introduction

Acute oxalate nephropathy (1-3) may be an under-reported, yet important complication of malabsorption-inducing weight loss supplements (4,5). We report on a patient taking orlistat, a lipase inhibitor, who presented with acute kidney injury (AKI) due to oxalate deposition.

## 2. Case Presentation

**Figure 1:**
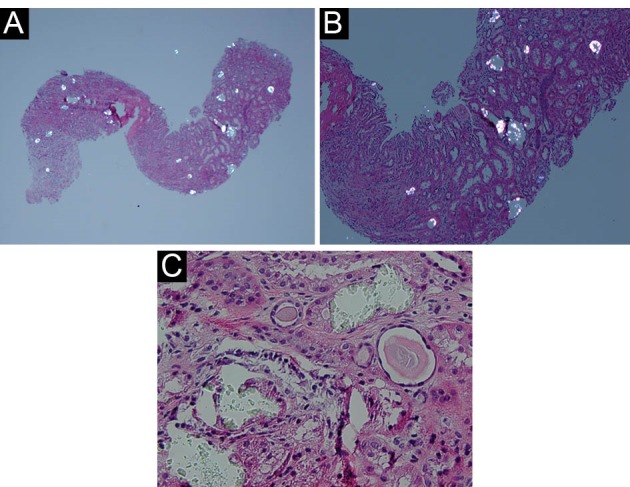
(A) Kidney biopsy tissue under low power, showing birefringent crystals under polarized light (×40, H&amp;amp;E). (B) Kidney biopsy tissue, showing birefringent crystals under polarized light (×100, H&amp;amp;E). (C) Kidney tissue biopsy under high power (×400, H&amp;amp;E).

Our patient was a 76-year-old white female with a past medical history of chronic kidney disease (CKD), baseline creatinine 1.85 mg/dL (estimated glomerular filtration rate 27 ml/min/1.73 m^2^; creatinine range 1.5-2.5 mg/dL six months prior to admission), hypertension, gout and psoriatic arthritis, who was admitted to the hospital for evaluation of elevated creatinine (4.83 mg/dL). She had no previous history of major surgeries. She did have a remote history of therapeutic use of arsenic for psoriasis approximately four decades earlier. She also had past history of heavy nonsteroidal anti-inflammatory drugs use up till three years earlier, when she had an episode of AKI. Her family history was unremarkable. She had only a remote history of smoking. Six weeks earlier, she has been started on orlistat 120 mg three times a day for weight loss. The rest of her medications included vitamin-C supplementation (500 mg/day) calcium carbonate, sevelamer hydrochloride and sodium bicarbonate supplementation for CKD, pantoprazole and monthly infliximab infusions for psoriatic arthritis. Physical exam was non-contributory, with a weight of 71 kg, height 1.55 m and blood pressure of 144/61 mm Hg. Her body mass index calculated at 29.5 kg/m^2^. Urinalysis with microscopy was unremarkable, except for 5 WBCs/high power fields with no crystals or cast formation. Extensive serologic work-up (antinuclear antibody, anti-neutrophil cytoplasmic antibodies, hepatitis-B and C studies, serum protein electrophoresis with measurements of serum free light chains) remained unremarkable. Uric acid was only mildly elevated at 7.2 mg/dL. Parathyroid hormone level returned within normal limits. During the diagnostic work-up, however, renal ultrasound noted multiple non-obstructing stones. Despite appropriate medical therapy, including volume expansion and correction of serum bicarbonate, creatinine rose to 5.40 mg/dL. Due to the ongoing diagnostic uncertainty a percutaneous kidney biopsy was performed, revealing calcium oxalate crystals within tubular lumens with associated interstitial inflammation with associated features of acute tubular necrosis (Figures 1A-C). A subsequent, 24-hour urine collection confirmed increased oxalate excretion (69.5 mg/24 hour; normal for the laboratory: 9.7 - 40.5 mg/24 hour specimen). Heavy metal screen (arsenic, cadmium, lead, mercury) from blood and 24-hour urine collection was unremarkable. Renal replacement therapy with intermittent hemodialysis was initiated for 5 consecutive sessions in eight days, which she tolerated well. Initial serum oxalate was 45 mm/l (normal <27, reporting limit > 10; ARUP Laboratories, Salt Lake City, UT/National Medical Services, Willow Grove, PA); subsequent values returned undetectable after renal dialysis begun. Repeated 24-hour urine collection before discharge documented ongoing excessive oxalate excretion (75 mg/24 hour) (Table 1.).

**Table 1:** Serum and urine biochemistry and clinical therapy review

Date	**Admission (09/2013)**	**Discharge (12 days after admission)**	**09/2013 (7 days after discharge)**	**10/2013**	**11/2013**	**02/2014**	**09/2014**	**05-06/2015**	**07-08/2015**
Creatinine, mg/dL	4.8	2.36	4.42	3.02*	2.9	2.28	2.04	2.30	2.32
Calcium, mg/dL	8.2	9.4	9.3	9.2	10.3^#^	9	9.5	9.6	9.6
Phosphorus, mg/dL	7.7	2.2	4.5	4.5	4.7^#^	4.7	4.1	3.4	n/a
Serum HCO3^-^. mM/L	18	29	22	25	26	22	26	26	29
Oxalate binder therapy		CaCO_3_*per os* 1250 mg, x3/day	CaCO_3_*per os* 1250 mg, x3/day	CaCO_3_*per os* 1250 mg, x3/day	CaCO_3_*per os* 1250 mg, x3/day	CaCO_3_*per os* 1250 mg, x3/day	Calcium citrate *per os* 1040 mg, x3/day	Calcium citrate *per os* 1040 mg, x3/day	Calcium citrate *per os* 2080 mg, x3/day^##^
Timed urine studies/24 hours
Urine creatinine, gm/24	0.61**		1.39	0.94*	1		1.31	1.39	0.85
Urine volume, L	0.812		5.2	3.8	3		4.95	4.85	2
Urine oxalate, mg/24 hour (normal: 9.7-40.5 mg)	54.6		75	96	54			52	31
Urine citrate, mg/24 hour	92			95	111			160	244
Urine calcium, mg/24 hour (normal: 100-300 mg)	8		36		45				60
Protein, mg	211								Non-measurable (creatinine < 4 mg/dL)

*measured creatinine clearance 24.3 cc/min, **performed during admission (09/13-15/2013), #on daily calcitriol ,##also on NaHCO_3_ 650 mg x3/day. To convert creatinine from mg/dL to µmol/l, multiply it by 88.4.

During follow-up, despite good medical compliance, she failed her *per os* calcium-carbonate therapy to achieve effective gastrointestinal oxalate chelation. Thereafter, being aware of the potential interaction between proton pump inhibitor (PPI) and reduced bioavailability of CaCO_3_ (6,7), she was changed to calcium citrate 1040 mg three times daily with meals for the purposes of gastrointestinal oxalate binding agent and to decrease urinary oxalate excretion (8). Twenty-two months after her initial hospital presentation she continues to do well and serum creatinine gradually decreased to the 2.01–2.32 mg/dL range (estimated glomerular filtration rate 20-24 ml/min/1.73 m^2^) ( Table 1). Urine oxalate excretion came under acceptable control with *per os* calcium-citrate dosed at 2080 mg, to be taken three times a day with meals ( Table 1).

## 3. Discussion

Obesity is a major world-wide epidemic linked to a number of chronic health risks such as heart disease, diabetes and high blood pressure (9,10).It has been reported to affect about one-third of the American adult population (9) and a recognized risk factor for kidney disease (10). Orlistat is a gastrointestinal lipase inhibitor used as an adjunct treatment of obesity and type 2 diabetes mellitus, to induce clinically significant weight loss by causing fat malabsorption.Malabsorption of intestinal lipids would, however, lead to increased “saponification” of calcium in the gastrointestinal tract and decreases calcium availability to form insoluble calcium oxalate complexes. The decreased binding of oxalate will lead to excessive oxalate absorption and will also appear in the urine. Due to the low solubility of oxalate, increased concentrations of oxalate in the body can lead deposition of calcium oxalate in the kidney tissue resulting in nephrocalcinosis, nephrolithiasis, and ultimately progressive renal insufficiency1. Both anti-obesity (bariatric) surgery (11) and orlistat administration is known to increase urinary appearance of oxalate (12). Acute oxalate nephropathy (AON) is defined as renal insufficiency in the presence of calcium oxalate crystal deposition in the renal interstitium and renal tubular cells (4,5). Currently, there is very limited data reported regarding orlistat-induced AON in the United States. The first case reported in 2007 described a patient with AON with a temporal relationship to an increased dose of orlistat and the development of increased fat malabsorption (more frequent loose oily stools) (4). Additional case reports have been described since (5,13,14). More comprehensively, a Canadian study of 953 patients reviewed the incidence of AKI twelve months before and after starting orlistat (15).The incidence of AKI twelve months before was 5 cases and 18 cases after. Our case, similar to past reported experience (16), also documented co-existing acute tubular necrosis (ATN) along with the crystal deposition. ATN is a common finding on renal biopsies when an acute rise of creatinine is documented in sick inpatients (17,18). Oxalate depositions are very common in kidney biopsies immediately after renal transplant and dialysis patients are known to have markedly elevated serum and tissue oxalate content (19,20). In our case, early initiation of temporary renal replacement therapy may have contributed to the excellent functional recovery. Other potential cause of oxalate deposition in this case was the intake of vitamin-C, which not an uncommon in complementary and alternative medicine and is also known precursor of oxalate (21,22). However, she has been taking her vitamin-C supplements already for years at the same dose unchanged. Ethylene glycol exposure may result in similar presentation (23), but she had no history of antifreeze exposure and serum anion gap was not elevated on admission. Unlike some of past cases (13), our patient did have a persistently elevated urinary oxalate excretion, even after cessation of orlistat therapy. It is uncertain, whether some or all of the reported individuals in the reported literature to date had an underlying mild or partial enzyme deficiency of alanine: glyoxylate aminotransferase, further aggravated by orlistat administration. Further, it is unclear, whether calcium supplement, originally intended as phosphorus binders do also reduce oxalate absorption and serum oxalate levels to a meaningful degree in advanced (stage 4-5) CKD patients. AON may be an under-recognized and important cause of renal failure in patients taking fat malabsorption-inducing weight loss supplements through hyperoxaluria.

## 4. Conclusions

Acute oxalate nephropathy is an important entity to recognize in patients taking weight loss supplements. If recognized early, acute oxalate nephropathy can be prevented and even may be reversible with discontinuation of offending agent and dietary modifications, including to provide effective gastrointestinal oxalate binders. Further, our case underscores the importance of kidney biopsy to facilitate early diagnosis and treatment.

## Acknowledgements

Parts of this paper has been presented in a poster format in National Kidney Foundation 2014 Spring Clinical Meeting, April 22 - 26, 2014, Las Vegas, NV. Am J Kidney Dis. 2014 (Apr); 63(5):A17

## Authors’ contribution

YH; first author, initial draft, correspondence, nephrology care of the patient. KCB; case identification, clinical correlation, general internal medicine care of the patient, review of manuscript. JRL; pathology correlations, review of the manuscript. AAL; critical review, literature.TF; senior author, literature, critical review, coordinating of manuscript revisions, nephrology care of the patient.

## Conflicts of interest

The authors declared no competing interests.

## Funding/Support

No special source of funding.

## References

[1] Nasr SH, D’Agati VD, Said SM, Stokes MB, Largoza MV, Radhakrishnan J (2008). Oxalate nephropathy complicating Roux-en-Y gastric bypass: an underrecognized cause of irreversible renal failure. Clin J Am Soc Nephrol.

[2] Mascio HM, Joya CA, Plasse RA, Baker TP, Flessner MF, Nee R (2015). An unusual cause of acute kidney injury due to oxalate nephropathy in systemic scleroderma. Clin Nephrol.

[3] Rankin A, Walsh S, Summers S, Owen M, Mansell M (2008). Acute oxalate nephropathy causing late renal transplant dysfunction due to enteric hyperoxaluria. Am J Transplant.

[4] Singh A, Sarkar SR, Gaber LW, Perazella MA (2007). Acute oxalate nephropathy associated with orlistat, a gastrointestinal lipase inhibitor. Am J Kidney Dis.

[5] Karamadoukis L, Shivashankar G, Ludeman L, Williams A (2009). An unusual complication of treatment with orlistat. Clin Nephrol.

[6] O’Connell MB, Madden DM, Murray AM, Heaney RP, Kerzner LJ (2005). Effects of proton pump inhibitors on calcium carbonate absorption in women: a randomized crossover trial. Am J Med.

[7] Targownik LE, Lix LM, Metge CJ, Prior HJ, Leung S, Leslie WD (2008). Use of proton pump inhibitors and risk of osteoporosis-related fractures. Can Med Assoc J.

[8] Harvey JA, Zobitz MM, Pak CY (1985). Calcium citrate: reduced propensity for the crystallization of calcium oxalate in urine resulting from induced hypercalciuria of calcium supplementation. J Clin Endocrin Metab.

[9] Ogden CL, Carroll MD, Kit BK, Flegal KM (2014). Prevalence of childhood and adult obesity in the United States, 2011-2012. JAMA.

[10] Chandra A, Biersmith M, Tolouian R (2014). Obesity and kidney protection. J Nephropathol.

[11] Sinha MK1, Collazo-Clavell ML, Rule A, Milliner DS, Nelson W, Sarr MG (2007). Hyperoxaluric nephrolithiasis is a complication of Roux-en-Y gastric bypass surgery. Kidney Int.

[12] Sarica K, Akarsu E, Erturhan S, Yagci F, Aktaran S, Altay B (2008). Evaluation of urinary oxalate levels in patients receiving gastrointestinal lipase inhibitor. Obesity.

[13] Chaudhari D, Crisostomo C, Ganote C, Youngberg G (2013). Acute oxalate nephropathy associated with orlistat: a case report with a review of the literature. Case Reports Nephrol.

[14] Coutinho AK, Glancey GR (2013). Orlistat, an under-recognised cause of progressive renal impairment. Nephrol Dial Transplant.

[15] Weir MA, Beyea MM, Gomes T, Juurlink DN, Mamdani M, Blake PG (2011). Orlistat and acute kidney injury: an analysis of 953 patients. Arch Intern Med.

[16] Karamadoukis L, Ludeman L, Williams AJ (2008). Is there a link between calcium oxalate crystalluria, orlistat and acute tubular necrosis?. Nephrol Dial Transplant.

[17] Tavares MB, 
Chagas de Almeida Mda
 
C
, Martins RT, de Sousa AC, Martinelli R, dos-Santos WL (2012). Acute tubular necrosis and renal failure in patients with glomerular disease. Ren Fail.

[18] Fülöp T, Alemu B, Dossabhoy NR (2014). Safety and Efficacy of Percutaneous Renal Biopsy by Physicians-in-Training in an Academic Teaching Setting. South Med J.

[19] Hoffman GS, Schumacher HR, Paul H, Cherian V, Reed R, Ramsay AG (1982). Calcium oxalate microcrystalline-associated arthritis in end-stage renal disease. Ann Int Med.

[20] Costello JF, Sadovnic MJ, Cottington EM (1991). Plasma oxalate levels rise in hemodialysis patients despite increased oxalate removal. J Am Soc Nephrol.

[21] Baxmann AC, Mendonça CD, Heilberg IP (2003). Effect of vitamin C supplements on urinary oxalate and pH in calcium stone-forming patients. Kidney Int.

[22] Levine M, Conry-Cantilena C, Wang Y (1996). Vitamin C pharmacokinetics in healthy volunteers: evidence for a recommended dietary allowance. Proc Natl Acad Sci U S A.

[23] Alhamad T, Blandon J, Meza AT, Bilbao JE, Hernandez GT (2013). Acute kidney injury with oxalate deposition in a patient with a high anion gap metabolic acidosis and a normal osmolal gap. J Nephropathol.

